# Recent Advances in the Discovery of Biomarkers for Canine Osteosarcoma

**DOI:** 10.3389/fvets.2021.734965

**Published:** 2021-10-01

**Authors:** Anita K. Luu, Geoffrey A. Wood, Alicia M. Viloria-Petit

**Affiliations:** ^1^Department of Biomedical Sciences, Ontario Veterinary College, University of Guelph, Guelph, ON, Canada; ^2^Department of Pathobiology, Ontario Veterinary College, University of Guelph, Guelph, ON, Canada

**Keywords:** canine osteosarcoma, biomarker, liquid biopsy, personalized medicine, microRNA (miRNA), extracellular vesicles, tissue biopsies

## Abstract

Canine osteosarcoma (OSA) is an aggressive malignancy that frequently metastasizes to the lung and bone. Not only has there been essentially no improvement in therapeutic outcome over the past 3 decades, but there is also a lack of reliable biomarkers in clinical practice. This makes it difficult to discriminate which patients will most benefit from the standard treatment of amputation and adjuvant chemotherapy. The development of reliable diagnostic biomarkers could aid in the clinical diagnosis of primary OSA and metastasis; while prognostic, and predictive biomarkers could allow clinicians to stratify patients to predict response to treatment and outcome. This review summarizes biomarkers that have been explored in canine OSA to date. The focus is on molecular biomarkers identified in tumor samples as well as emerging biomarkers that have been identified in blood-based (liquid) biopsies, including circulating tumor cells, microRNAs, and extracellular vesicles. Lastly, we propose future directions in biomarker research to ensure they can be incorporated into a clinical setting.

## Introduction

Osteosarcoma (OSA) is an aggressive neoplasm of the bone that is characterized by the production of osteoid (1). In canines, OSA accounts for 85% of tumors that arise in the bone and has an incidence rate of 13.9/100,000 per year, compared to 1.2/100,000 per year in humans (2, 3). Large breed (>40 kg) dogs that are middle-age and older are most affected, with a median age at diagnosis of 7 years (4). Most cases present in the appendicular skeleton, with the forelimb being more commonly impacted than the hindlimb. Dogs often present with lameness, swelling at the site, and pain. Diagnosis is based on a physical examination and radiographs of the lesion. Pre-operative histological analysis of the lesion offers a definitive diagnosis and can be performed on bone biopsies obtained through open incisional, closed needle or trephine biopsy techniques (1, 5). However, this procedure is quite invasive and increases the risk of pathological fracture. As such, fine needle aspirate of the bone lesion offers a less invasive diagnostic alternative (6–8). The initial work-up also involves thoracic radiographs to determine the presence of lung metastasis (9).

Treatment for canine OSA involves removal of the primary tumor through either limb-spare or limb amputation surgery. Patients that undergo surgery alone have a short median survival time of 101–177 days (~6 months) due to the development of lung metastases, the most common site of OSA metastasis (10–14). Other reported metastatic sites include bones, lymph nodes, skin and subcutaneous tissues (4, 15, 16). Although most canine OSA patients do not present with radiographically detectable metastatic disease at diagnosis, ~90–95% of canine OSA patients have micrometastases as is evident through the formation of macrometastases after removal of the primary tumor (17). Adjuvant chemotherapy treatment with carboplatin, cisplatin and doxorubicin post-surgery has been helpful in extending the median survival time to ~247–366 days, depending on the agent used (10, 11, 13, 14, 18–24). Despite this treatment approach, a majority of OSA patients will develop metastasis, a clinical problem in both human and canine OSA (25, 26).

## Potential Utility of Biomarkers

**Figure 1:**
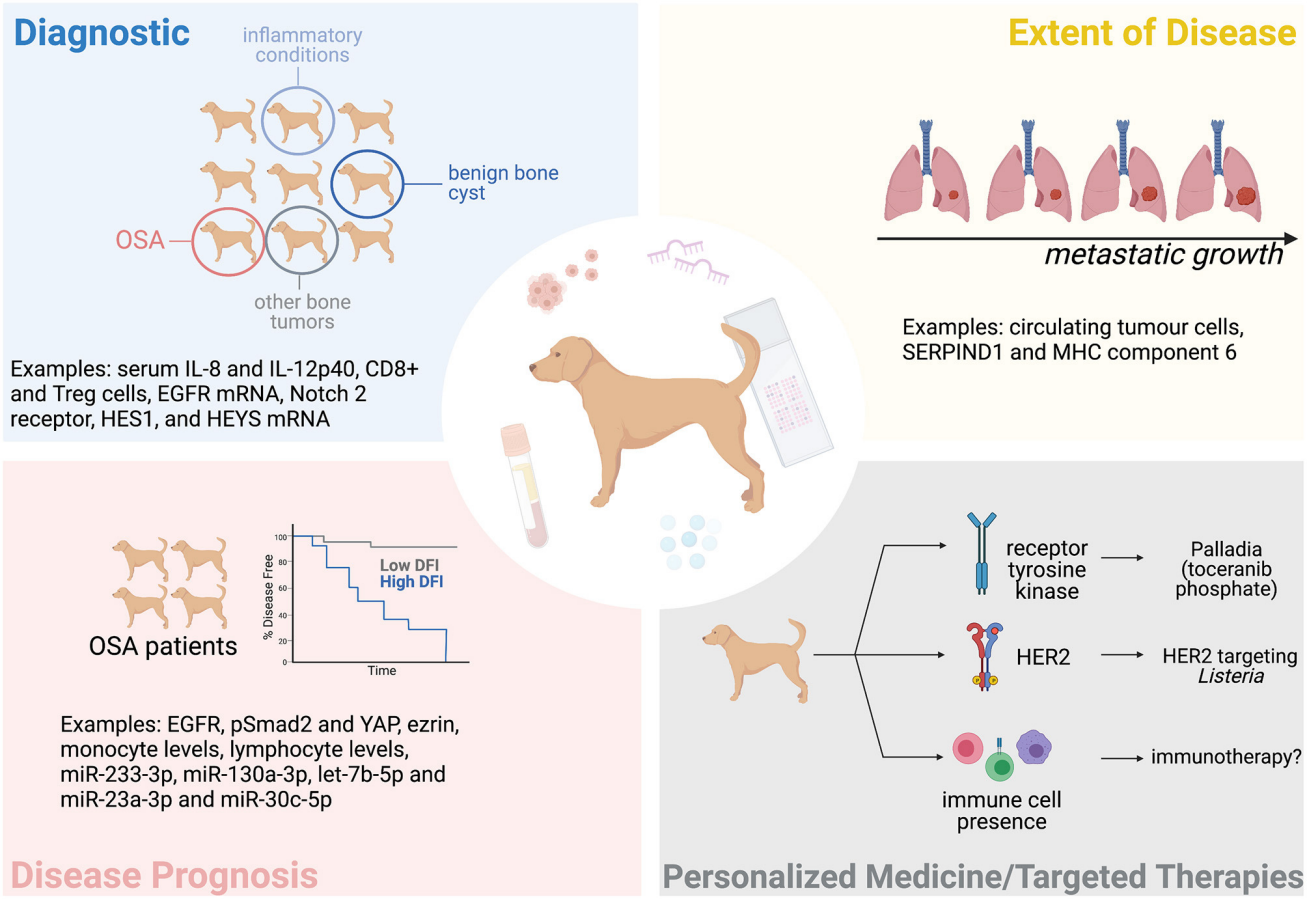
The potential of biomarkers in the clinical management of canine OSA. Tissue and blood-based (liquid) biopsies could help diagnose OSA, determine the extent of a patient's disease (metastatic progression), the patient's prognosis, and help personalize treatment. Some examples mentioned in this manuscript are indicated here. Figure created with BioRender.com.

The variability in the time it takes dogs to develop detectable metastasis after treatment, as well as variability in the overall survival (OS) time, suggests that patients respond differently to treatment; yet current practice effectively lumps all these patients into the same category of “poor prognosis.” Biomarkers could be particularly informative in this regard and are defined by the NIH Biomarkers Definitions Working Group as a “characteristic that can be objectively measured and evaluated as indicator of biological processes, pathogenic processes, or pharmacologic responses to a therapeutic intervention” (27). Biomarkers could be used as a diagnostic tool, to determine the extent of disease, to indicate disease prognosis, and to predict or monitor the clinical response to a particular intervention (Figure 1). This could benefit both the client and clinician as it would better predict a patient's disease course, allowing more informed decisions. Biomarkers could also specify the molecular features of a tumor and potentially permit a personalized treatment approach.

Alkaline phosphatase (ALP) status, body weight, and location of the primary tumor are examples of proposed biomarkers. Greater weight at the time of diagnosis has been associated with metastasis; while primary lesions in the proximal humerus, distal femur, or proximal tibia and age are associated with an increased mortality (28). Two grading systems have also been developed by Kirpensteijn et al. (29) and Loukopoulos and Robinson (30), which was modified based on features described by Straw et al. (31). However, when both systems were evaluated in 85 canine appendicular OSA tumor samples that received standard of care (SOC) treatment (amputation and adjuvant chemotherapy), neither were effective at predicting disease outcomes (32). This lack of clinically relevant biomarkers may reflect the biological complexity of OSA, but can also be attributed to underpowered studies, lack of consistent treatment and other inclusion criteria, and poor-quality data on time of actual metastasis vs. euthanasia due to comorbidities such as cardiovascular and respiratory disease or osteoarthritis (33). This review summarizes literature that has explored the diagnostic and prognostic use of various tissue-based and blood-based (liquid biopsy) markers, see Table 1.

**Table 1:** Summary of biomarkers explored in canine appendicular OSA using tissue and blood-based (liquid) biopsies.

**Molecule/factor**	**Diagnostic**	**Disease-free interval (DFI)**	**Overall survival (OS)**	**Other**	**References**
**Tissue biopsies**					
EGFR	High levels of EGFR mRNA in OSA compared to normal bone	Higher levels of EGFR protein associated with shorter DFI	Higher levels of EGFR protein associated with shorter OS		(34)
B catenin		Moderate/high cytoplasmic protein levels associated with metastasis (35); No association found in (36)	No association with survival (36)	No difference in B catenin mRNA and protein levels between patients with high vs. normal ALP (37)	(35–37)
HER2	40% of canine OSA tissue had higher levels of HER2 mRNA than normal bone		HER2 negative tumors had 115-day shorter survival time		(38)
PDGFalpha and beta/PDGFRalpha and beta		No association	No association		(39)
Notch/HES1	High levels of Notch 2 receptor, HES1, and HEYS mRNA in bone tumor samples compared to normal bone	HES1 mRNA higher patients with DFI > 300; Low levels of HES1 protein associated with shorter DFI			(40)
TAZ/YAP and pSmad2		High levels of pSmad2 and YAP immunolabelling associated with earlier metastasis	High levels of pSmad2 and YAP immunolabelling associated with shorter survival; no associations with mRNA levels		(41)
IGFR1			High levels associated with shorter OS		(42)
PTHR1/PTHrP			High levels of PTHR associated with survival; not significant for PTHrP		(43)
TP53			Moderate/high nuclear TP53 associated with shorter OS		(35)
Survivin		Nuclear survivin correlated with longer DFI	High immunoreactivity predicted shorter DFI (44) Nuclear survivin correlated with longer OS	Nuclear survivin correlated with caspase 3	(35)
CDKN2A (P16)		Presence of P16 staining had no associations with metastasis	Presence of P16 staining trending toward a shorted survival time (not significant)		(45)
Ezrin		High levels of Ezrin associated with shorter DFI			(46)
CD204^+^ macrophages		High CD204^+^ macrophages in tumor tissue associated with improved DFI (47)		CD204^+^ macrophages are higher in pulmonary metastases (48)	(47, 48)
CD3^+^ and FOXP3^+^ cells				CD3^+^ lymphocytes and FOXP3^+^ cells in primary OSA tissue correlated with those in metastatic lesions; T- and B- lymphocytes are higher in pulmonary metastases	(48)
miR-233-3p, miR-130a-3p, let-7b-5p		miR-223-3p, miR-130a-3p, and let-7b-5p was able to determine high DFI risk (123.5 days) from low DFI risk (392 days)			(49)
**Blood-based (liquid) biopsies**					
**Whole blood**					
Monocytes		>0.4 ×10^3^ monocytes/μL before treatment associated with shorter DFI (50)	> 0.4 ×10^3^ monocytes/μL doesn't predict survival	Monocytes decrease CCR2 and CXCR2 levels	(51)
Lymphocytes		> 1.0 ×10^3^ lymphocytes/μL before treatment associated with shorter DFI			(52)
CD8^+^ and Treg cells	OSA patients have lower CD8^+^ cells, and higher Treg cells compared to healthy dogs		Low CD8^+^/Treg ratio associated with shorter OS		(53)
Circulating tumor cells		CTCs in peripheral blood drops after amputation, and increases before metastases or euthanasia			(54)
**Serum**					
Extracellular vesicles	10 proteins higher in serum EVs from OSA patients, compared to serum EVs from normal and traumatic fracture samples	SERPIND1 and MHC component 6 higher in serum EVs from OSA patients with progressive disease compared to serum EVs at diagnosis and post-amputation			(55)
Extracellular vesicles		Tetranectin could discriminate good responders (DFI > 300) from poor responders (DFI <100); Complement C2 and C3, alpha-2-macroglobulin and protein S could discriminate poor responders (DFI <100) from good responders (DFI >300)			(56)
IL-8 and IL-12p40	IL-8 and IL-12p40 significantly higher in serum samples of canine OSA patients compared to healthy controls at time of diagnosis				(57)
miR-23a-3p and miR-30c-5p		miR-23a-3p and miR-30c-5p separated patients by high risk DFI (123.5 days) from low risk DFI (272 days)			(49)
**Plasma**					
miR-214 and miR-126	miR-214 and−126 high in OSA; miR-214 might be more specific to sarcomas. miR-214 and−126 have diagnostic potential (95.5% specificity) in sarcomas when combined	High levels of miR-214 associated with shorter DFI for 1- and 6-year outcomes; high miR-126 associated with prolonged DFI	High levels of miR-214 associated with OS for 1- and 6-year outcomes; high miR-126 associated with prolonged OS		(58, 59)
TGFβ1				Associated with Urine N-telopeptide; levels of TGFβ1 decrease after treatment	(60)

## Tissue-Biopsy Based Markers

Molecular profiling of tumor tissue is an invaluable way to understand what signaling pathways and genes may be contributing to cancer progression. Gene expression microarray analysis found 51 genes to be differentially expressed between short survivors (<6 months) and long survivors (≥6 months). A majority of these genes were upregulated in the short survivors group and mediate drug resistance, proliferation, or metastasis. Pathways that were overactive in short survivors included Wnt, chemokine/cytokine, and integrin signaling (61). A similar analysis was completed by O'Donoghue et al., but they explored gene expression differences for dogs with disease free interval (DFI) <100 days, and > 300 days (62). Pathway analysis identified genes involved in cell adhesion, cytoskeletal remodeling, immune response, cAMP/protein kinase A (PKA) signaling, oxidative phosphorylation and bone development to be differentially expressed. Given the role of these pathways and cellular processes in promoting OSA progression, related molecules have been investigated in subsequent studies at the mRNA and protein level (see below) and found to correlate with patient outcomes.

### Cell Signaling Pathways

The hyperactivation of several pathways have been suggested to promote the growth of OSA cells. The increased expression of receptor tyrosine kinases (RTK) can lead to enhanced downstream signaling and pro-tumourigenic phenotypes. OSA tumors have significantly higher levels of epidermal growth factor receptor (EGFR) mRNA compared to normal bone tissue. Analysis in a tissue microarray (TMA) found that patients with high levels of EGFR protein had shorter OS and DFI, compared to patients with low or negative levels (34). In the same family of EGFR is human epidermal growth factor receptor-2 (HER2 or ErbB2), a well-known proto-oncogene in mammary carcinomas. A small study involving 10 canine OSA tissue samples found that HER2 is significantly overexpressed in 40% of tumors compared to their normal counterparts. The sample size was not conducive for a robust statistical analysis in terms of patient outcome, but the authors noted that dogs that were HER2 negative had a 115-day longer survival time post-amputation (38). However, the antibody used to detect HER2 in this study is discontinued and there was no validation of specificity to canine HER2 conducted, so it is not clear that HER2 protein is expressed in canine OSA. Indeed, specificity of antibodies raised against human proteins and applied to canine proteins has been an issue in other studies. Burrai et al. tested a different HER2 antibody in canine mammary cancers and found unexpected cytoplasmic immunolabelling on immunohistochemistry (IHC), several bands of inappropriate molecular weights on Western blots, and no HER2 detection by mass spectrometry in any canine mammary tumors that were positive on IHC (63).

Insulin growth factor receptor 1 (IGFR1) and platelet derived growth factor (PDGF) signaling promote cancer cell proliferation and survival by activating PI3K/AKT and mitogen-activated kinase (MAPK) signaling. High levels of IGFR1 were found to be associated with reduced OS but no association was observed between IGFR1 levels and DFI (42). Neither PDGF ligands nor receptors were found to associate with DFI or OS (39). Notch signaling, mediated by a non-kinase receptor, has also been examined in canine OSA. Tumor tissue has increased mRNA expression of Notch2, and downstream mediators hairy ears, Y-linked (HEY), and hairy and enhancer of split 1 (HES1) compared to normal bone. However, when comparing patients with DFI <100 and DFI > 300 days, HES1 mRNA was significantly higher in the DFI > 300 cohort. This trend was also seen at the protein level, as higher HES1 protein immunoreactivity associated with a longer DFI (40).

### Bone Development Pathways

Wnt signaling is involved in bone development, and its overactivation has been observed in OSA (64). Stein et al. found that a majority of canine primary OSA tumors were positive for β-catenin, but found no association with patient outcome (36). Others observed that β-catenin mRNA and protein levels did not differ between patients with elevated vs. normal ALP levels (37). The localization of β-catenin may be more reflective of OSA aggressiveness, as moderate/high levels of cytoplasmic β-catenin was predictive of metastasis in another study (35). Other well-known mediators of bone development are transforming growth factor beta (TGFβ) and Hippo signaling (65–67). Our findings suggest that TGFβ and Hippo signaling cooperate to promote OSA progression *via* activated SMAD2 and yes-associated protein (YAP), as high levels of phosho-SMAD2 and YAP in canine OSA tissue associated with reduced DFI and OS (41). Parathyroid hormone receptor 1 (PTHR1) is another known regulator of bone formation (68) and functions through cAMP/PKA signaling (69), the activity of which was shown to mediate OSA development in a p53 mutant model of OSA (70). High levels of PTHR1 in canine OSA tissue was associated with decreased OS (43).

### Tumor Suppressors, Cell Cycle, and Cell Survival Mediators

The levels and localization of tumor suppressors and cell cycle mediators have also been explored in OSA tumors. The protein levels of TP53, PTEN, RB1, and CDKN2A (P16), which were previously found to be mutated in OSA, were assessed in a canine OSA TMA comprised of 150 cases (71). Differences in localization (nuclear, cytoplasmic or both), staining positivity (presence of staining), and staining intensity (darkness of staining) were demonstrated between tissue cores, although associations with outcome were not explored. Another study found that patients with high nuclear TP53 expression have a shorter OS (35). P16 expression was also evaluated in another study but did not consider localization. Though not statistically significant, patients with tumors with any immunoreactivity to P16 trended toward a shorter OS, compared to patients whose tumors had no immunoreactivity (45). To increase cell division and survival, cancer cells may upregulate the expression of survivin, an inhibitor of apoptosis (72). Surprisingly, a significant positive correlation was found between nuclear survivin levels and pro-caspase 3 levels, and DFI and OS (35). These results contrast those from another study that found patient with high levels of survivin immunoreactivity had a shorter median DFI of 173 days, compared to 331 days when immunoreactivity was low (44).

### Immune Response

Previous studies have shown that developing an infection post-limb-spare surgery is a positive predictor for recurrence and survival, suggesting that an effective immune response could provide an anti-tumor effect (73, 74). However, this association between wound site infection and survival was not seen in patients undergoing amputation (75).

When considering dogs that had undergone limb-amputation and chemotherapy, high levels of CD204^+^ macrophages within the primary tumor associated with a longer DFI (47). This is an interesting finding given that CD204 is considered a marker of M2 tumor promoting macrophages, whose enrichment associates with poor prognosis in some epithelial tumors (76, 77). To determine how immune cell populations differ between the primary and metastatic lesion, Withers et al. compared the expression of various immune cell markers in 21 paired samples. It was found that CD3^+^ (T-lymphocytes), and FOXP3^+^ (T-regulatory) cells were positively correlated between the two sites. To determine if there were significant differences in immune cell infiltrates between the primary and metastatic lesion, CD3^+^, PAX-5 (B cell) and CD204^+^ (macrophage) levels were compared. All three cell types were significantly increased at the metastatic lesion, compared to the primary (48). These results suggest that the primary tumor could provide indication of the immune environment at the metastatic lesion. It should be noted that almost all the metastatic lesions were taken at necropsy. Thus, these results only represent advanced metastases, complicating their potential use in guiding immunotherapy approaches in the micrometastasis setting.

### Metastasis

Metastasis is a complex process, and its success depends on many different factors [recently reviewed in (78)]. Of the proteins proposed to play a role in OSA metastasis, the cytoskeleton-to-membrane molecule, ezrin, is the best characterized. High ezrin expression in the primary tumor is an indicator of a short DFI, most likely due to ezrin's ability to facilitate lung colonization (46). Matrix metalloproteinases-2 and−9 (MMP-2/-9) are also expressed in primary OSA tissue, but no associations with outcome has been reported (50, 79, 80).

The aforementioned studies demonstrate the importance of various molecules in OSA progression in a patient relevant context and provide justification for the development and use of certain targeted therapies [i.e., tyrosine kinase receptor inhibitors (81, 82) and HER2-targeting Listeria (83)]. However, tissue biopsies only provide a static view of the tumor and do not account for tumor heterogeneity and evolution. Liquid biopsies could be particularly advantageous in this regard.

## Blood-Based (Liquid) Biopsies

Liquid biopsies allow for the dynamic evaluation of pathological processes in bodily fluids such as blood and urine. They offer some advantages over tissue biopsies as they are minimally invasive and allow for serial monitoring throughout the course of a patient's treatment [see review in (84) for more comparisons]. This could be helpful in OSA since most canine patients undergo limb amputation, and tissue samples from secondary lung lesions are not easily accessible. As OSA predominately metastasizes *via* the blood stream (85), and blood serves as a conduit for both cells and cell products, many of the studies focus on blood as the source. The section below summarizes the limited literature in this field.

### Immune Cells and Immune Cell Modulators

The blood contains a plethora of cells which can provide insight on the disease status of a patient. As complete blood counts are routinely completed during medical visits, evaluating the quantity of immune cells in circulation could be informative and a feasible way to monitor a patient over time. Sottnick et al. observed that high levels of monocytes (>0.4 × 10^3^/μL) and lymphocytes (> 1 × 10^3^/μL) before SOC treatment was associated with a significantly shorter DFI (52). This >0.4 × 10^3^/μL monocyte cut off was unfortunately unable to predict OS in another study, possibly due to different inclusion criteria of patient cohorts (51).

When evaluating T lymphocytes specifically, it was found that the number of CD8^+^ cells was significantly lower, while the number of T regulatory cells was significantly higher in the blood of canine OSA patients compared to healthy controls. When considering both cell populations together, a low CD8^+^/T regulatory cell ratio was associated with a significantly shorter OS (53).

Aside from levels and ratios of specific number of immune cells, the levels of specific cytokines may also be informative. A preliminary study showed that serum levels of IL-8 and IL-12p40 was significantly higher in canine OSA patients compared to healthy controls (57). The plasma levels of TGFβ1, one of the three ligands that activate TGFβ signaling, was demonstrated to correlate with urine N-telopeptide, a marker of bone resorption that is significantly higher in OSA patients (86). TGFβ1 plasma levels were also lower after ionizing radiation and zolendronate treatment, suggesting that TGF β1 may be a good marker of bone resorption (60).

### microRNAs

microRNAs are non-coding RNAs that are typically 18–25 nucleotides in length. miRNAs bind to target mRNAs to induce their degradation or inhibit their translation, thus modulating protein levels and cell behavior (87). miRNAs can be useful as circulating biomarkers as they enter circulation inside extracellular vesicles, like exosomes (88), and by forming complexes with proteins like Argonaute2 (89, 90).

Heishima et al. found that miR-214 and miR-126 are significantly elevated in the plasma of canine sarcoma patients when compared to controls. These microRNAs may be particularly relevant in OSA, as circulating levels were deemed high when compared to other malignancies explored in the study. Thus, miR-214 and miR-126 could potentially be a sensitive and accurate diagnostic marker when considered in combination, but this will have to be confirmed for OSA specifically (58). A later follow-up study evaluated miR-214 and miR-126 levels in the pre-treatment plasma of canine OSA patients receiving SOC. Univariate analysis found that high miR-214 levels associated with a shorter DFI and OS for 1- and 6-year outcomes. The trend was not replicated for miR-126, as high levels were associated with a prolonged DFI and OS, but only when considering 1-year outcomes. An integrated analysis, which combined both miRNAs and serum ALP status, was more effective at predicting DFS and OS compared to ALP alone (59). A recent study evaluated miRNA expression in canine OSA tumor tissue and serum to identify associations with patient outcome. A three-miRNA model, miR-233-3p, miR-130a-3p, and let-7b-5p, was able to predict high risk (DFI = 123.5 days) from low risk (DFI = 392 days) patients when evaluated in tumor tissue. Serum miR-23a-3p and miR-30c-5p combined were also identified to have prognostic value and separated high risk (DFI = 123.5 days) from low risk (DFI = 272 days) patients (49). These are the only studies that reported associations between circulating miRNAs and outcome in canine OSA. Another study explored a miRNA-associated signature in OSA tumor tissue. It found that patients with an increased expression of a gene signature associated with miR-382 had a shorter OS than those that had a decreased expression (91).

### Circulating Tumor Cells

CTCs are cells released from the primary lesion, either through active intravasation or passive shedding. CTCs can then enter the blood stream individually, or in clumps, where they have to survive and eventually arrest at metastatic sites or get stuck in capillary beds (92). The appearance of CTCs in the peripheral blood of canines with metastatic mammary carcinoma is associated with poor survival outcomes (93).

In OSA, Wright et al. demonstrated that CTCs can be detected in the blood by flow cytometry. A majority of canine OSA CTCs are positive for collagen I (COLI) and weakly positive for osteocalcin (OCN). In an analysis of 3 patients, it was found that the number of CTCs declined after amputation but increased before metastatic disease or euthanasia (54).

### Extracellular Vesicles

Extracellular vesicles, or EVs, is a broad term that applies to membrane-bound vesicles released by cells into the extracellular environment. The cargo of EVs varies in biomolecule type and differs between cancers (94). These two features, as well as the high abundance of EVs in the blood, ~10^10^ EVs/1 mL of plasma (95), makes them extremely attractive cancer biomarkers.

To date, only two studies have explored the biomarker potential of circulating EVs in canine OSA. In one study, authors compared the protein content in a specific type of EV, known as exosomes, from the sera of dogs with OSA, dogs that suffered a fracture, and normal donors. From this analysis, 10 proteins were successfully able to distinguish OSA from the other groups with an 85% accuracy. To determine if the exosomal protein content changed throughout OSA progression, the sera of five dogs were compared pre-amputation, 2 weeks post-amputation and upon detection of progressive disease. SERPIND1 and major histocompatibility complex (MHC) class III component C6 were both able to distinguish the OSA by their various stages with 77% accuracy (55). In relation to outcome, a recent study evaluated exosomes in the sera of 10 canine OSA patients that were defined as poor responders (DFI <100 days) and good responders (DFI > 300 days). Exosomal tetranectin predicted a good prognosis, while the complement C2 and C3, alpha-2-macroglobulin and protein S predicted a poor prognosis (56). These results are encouraging but preliminary and warrant further investigation in larger patient cohorts.

## Looking Forward: Barriers to Overcome to Improve Clinical Applicability

The studies reviewed herein provided valuable information on OSA biology, but multiple barriers prevent the clinical application of the proposed biomarkers. Some of the studies compared only a handful of patients, leading to problems with statistical power (96). Almost all the findings described above are based on single studies, each using different quantification methods for their molecule of interest and creating different cut-offs for their desired clinical comparison. Altogether, this makes it extremely difficult to determine which biomarker candidates are most promising, extrapolate these findings to larger cohorts of OSA patients, and precludes their clinical use. Increased multi-institutional collaborative studies would increase patient recruitment (leading to larger sample sizes), determine the replicability of previous findings, and maximize the use of patient samples by screening multiple tissue-based and blood-based markers within the same patient.

The feasibility of evaluating some of the above markers in a clinical setting will also need to be improved. Isolating miRNA and EVs and detecting CTCs and blood cells require specialized equipment and reagents which makes the pipeline from sample acquisition to data acquisition cumbersome. Certain technologies, such as microfluidic devices (97) or synthetic peptides (98) would allow a more rapid isolation of EVs and multi-omic profiling (99). However, this will first require antibody validation or cross-species molecular approaches to permit the rapid detection of biomarkers in canine samples. These validation efforts need to also extend to antibodies used in cytokine studies. Human and canine cytokine amino acid sequences share 49–96% homology, but proper validation is necessary to confirm antibody cross-reactivity between species (100). Canine-specific cytokine assays may be advantageous as diagnostic tools but would also require appropriate and rigorous validation.

Given these obstacles, it is imperative that future biomarker research follows a critical discovery, validation, and clinical translation pipeline. The study populations included in the discovery and validation studies need to be well-documented, ideally with genetic background of the patient's tumor (given the chaotic nature of OSA), adequate history of treatments received, and complete dates of defined clinical endpoints. If the biomarker proves useful, focus should then be directed to developing technologies to allow its use in a clinic setting. As blood-based (liquid) biopsies are easier to access, permit serial sampling, and potentially have an easier clinical translation, future studies should focus on blood-based (liquid) biomarkers.

## Conclusion

Canine OSA lacks reliable diagnostic, prognostic, and predictive biomarkers. Several tissue-based and liquid-based biomarkers have been proposed in the literature. These studies showcase the potential of biomarkers to predict patient response to therapy, determine the presence of metastasis, predict patient outcomes, and allow for personalized medicine. Confirming the results in larger cohorts through collaboration and increasing the feasibility of evaluating these molecules in a diagnostic laboratory setting will bring biomarkers one step closer to becoming a clinical reality for canine OSA patients.

## Author Contributions

AL and AV-P conceived the ideas to develop the manuscript. AL drafted the manuscript and prepared all figures and tables. AV-P and GW revised the manuscript, figure, and table. All authors edited and approved the final manuscript.

## Funding

AV-P studies on canine OSA biomarkers are funded by OVC Pet Trust Grant #053666. GW studies on canine OSA biomarkers are funded by OVC Pet Trust Grant # 053527.

## Conflict of Interest

The authors declare that the research was conducted in the absence of any commercial or financial relationships that could be construed as a potential conflict of interest.

## Publisher's Note

All claims expressed in this article are solely those of the authors and do not necessarily represent those of their affiliated organizations, or those of the publisher, the editors and the reviewers. Any product that may be evaluated in this article, or claim that may be made by its manufacturer, is not guaranteed or endorsed by the publisher.
